# The effect of body mass index on neonatal outcomes in Chinese women with polycystic ovary syndrome

**DOI:** 10.3389/fmed.2022.996927

**Published:** 2022-11-14

**Authors:** Haiyan Guo, Bian Wang, Hongyuan Gao, Qianqian Zhu

**Affiliations:** Department of Assisted Reproduction, Shanghai Ninth People's Hospital Affiliated to Jiao Tong University School of Medicine, Shanghai, China

**Keywords:** body mass index, neonatal outcome, frozen embryo transfer, polycystic ovary syndrome, assisted reproductive technology

## Abstract

**Aim:**

This study aimed to explore the effect of body mass index (BMI) on neonatal outcomes in patients with polycystic ovary syndrome following the frozen embryo transfer (FET).

**Methods:**

This study included 1,676 singletons born from mothers with polycystic ovary syndrome (PCOS) after FET between 1 Jan 2007 and 31 Dec 2019. BMI was categorized into three groups: underweight (BMI less than 18.5 kg/m^2^), normal weight (BMI between 18.5 and 24.9 kg/m^2^), and overweight (BMI between 25.0 and 29.9 kg/m^2^). Logistic regression models with generalized estimating equations were used for clustering by patients to explore the effect of BMI on neonatal outcomes.

**Results:**

When compared to normal-weight mothers, the rate of large for gestational age (LGA) babies (adjusted odds ratio [aOR] 0.45, 95% confidence interval [95%CI] 0.22–0.93) significantly decreased for underweight mothers and significantly increased (aOR 1.82, 95%CI 1.38–2.41) for overweight mothers. The rate of high birth weight among infants from overweight mothers (aOR 1.75, 95%CI 1.15–2.65) was significantly higher than those from normal-weight mothers after adjusting for known confounding factors. The rate of small for gestational age (SGA) singleton (aOR 2.37, 95%CI 1.14–4.93) was lower among underweight mothers than normal-weight mothers.

**Conclusion:**

Maternal underweight was a significant protective factor against LGA infants for singletons born from patients with PCOS after FET, whereas maternal overweight was an adverse factor for LGA infants.

## Introduction

Polycystic ovary syndrome (PCOS) is one of the most common endocrine disorders affecting about 5–20% of reproductive-aged women (1). It is diagnosed based on the presence of oligo/anovulation, clinical and/or biochemical hyperandrogenism, and/or polycystic ovary morphology (2). PCOS presents reproductive manifestations (hyperandrogenism, anovulation, and infertility), metabolic implications (dyslipidemia, type 2 diabetes, and potential cardiovascular disease), and psychological problems (anxiety, depression, and poor self-esteem) causing heavy health and economic burdens, which has been considered an important clinical and public health issue (3–6).

Evidence shows that PCOS has a negative impact on neonatal outcomes. Infants born from mothers with PCOS are at increased risk of preterm birth, large for gestational age birth (LGA), admission to a neonatal intensive care unit, or perinatal mortality (7–10). Research has shown that pre-pregnancy maternal overweight or obesity is associated with poor infant outcomes such as LGA, macrosomia, and preterm delivery (11, 12). Overweight and obesity are common in women with PCOS, affecting 30–70% of the PCOS population, which may explain the increased risk of adverse neonatal outcomes in women with PCOS (13). Available studies of patients with PCOS assessing the influence of body mass index (BMI) on infant outcomes are performed in the general population (4, 14). A large proportion of women with PCOS presented with infertility and seek assisted reproductive technology (ART) to get pregnant (15). Consequently, it is unclear to what extent can the risk of adverse neonatal outcomes for women with PCOS undergoing ART treatment be attributed to abnormal BMI.

Fresh embryo transfer is the normal practice in many assisted reproductive centers for patients with PCOS. Following the rapid development of cryopreservation techniques, frozen embryo transfer (FET) is being used as a great substitute for fresh embryo transfer in clinical treatment. A multicenter randomized controlled trial among 1,508 infertile women with PCOS undergoing the first *in vitro* fertilization cycles reported that those with FET had a higher live birth rate and a lower risk of ovarian hyperstimulation syndrome than those with fresh embryo transfer (16). However, some research studies have been conducted to explore the influence of BMI on neonatal outcomes in patients with PCOS undergoing FET.

Therefore, this retrospective cohort study was performed to evaluate neonatal outcomes among underweight and overweight vs. normal-weight patients with PCOS following the FET.

## Materials and methods

### Study subjects

All data used in this retrospective study was extracted from the clinical database at the Assisted Reproduction center of the Shanghai Ninth People's Hospital (a large tertiary hospital in Shanghai, China). Cycle-level information on patient characteristics, details of clinical treatment with ART procedure, and pregnancy and neonatal outcomes were recorded in this database (17). A series of quality control practices were performed to ensure a high-quality database. Trained medical staff verified the diagnosis and checked the data and medical record review in accordance with standardized guidelines made by our center. The medical records of patients were randomly selected by surveillance staff for verification of the accuracy of the data. In this study, we included 1,676 singletons born from mothers with PCOS after FET during the period from 1 Jan 2007 to 31 Dec 2019. PCOS was diagnosed according to the revised diagnostic criteria of the Rotterdam consensus 2003 in this study, which required that at least two of the following three criteria were met: (1) oligo-or anovulation; (2) clinical and/or biochemical signs of hyperandrogenism; (3) polycystic ovaries and exclusion of other etiologies including congenital adrenal hyperplasia, androgen-secreting tumors, and Cushing's syndrome. Patients undergoing ART treatment and satisfying the above diagnostic criteria were included in this study. Patients with the following diseases were excluded: diabetes, hypertension, thyroid dysfunction, tumors, and chromosomal abnormalities.

### Procedure

Pre-pregnancy BMI was computed based on the weight and height recorded before the clinical treatment (weight (kg)/(height (m)^2^) and was divided into three categories according to the classification of the World Health Organization: underweight (BMI less than 18.5 kg/m^2^), normal weight (BMI between 18.5 and 24.9 kg/m^2^), and overweight (BMI between 25.0 and 29.9 kg/m^2^). In our center, the number of patients having a pre-pregnancy BMI of >30 kg/m^2^ was small; hence, we did not include these patients in this study. The following reasons can account for the small number of obese women. On the one hand, the BMI of the Asian population is generally lower than that of non-Asian populations (18). In addition, weight loss is often advised for obese infertile women before starting ART treatments according to international clinical recommendations (19). We used the normal weight as the reference.

For the neonatal outcome, clinical data of gestational age and birth weight were collected. The gestational age for cleavage-stage embryo transfer was calculated by pulsing 17 days since the date of embryo transfer, while the gestational age for blastocyst transfer was calculated by pulsing 19 days since the date of embryo transfer. Preterm birth was defined as the delivery before 37 completed gestational weeks by the World Health Organization, and gestational age of lesser than 32 weeks was categorized as very preterm birth. The birth weights of <2,500 and >4,500 g were categorized into the low birth weight group and the high birth weight group, respectively. LGA and SGA were defined as birth weight over the 90th percentile and below the 10th percentile for gestational age, respectively, according to the Chinese sex- and gestational age-specific birth weight standards (20). Appropriate for gestational age (AGA) was referred to as birthweight between the 10^th^ and 90^th^ percentiles for gestational age.

### Statistical analysis

The distributions of basic characteristics in different BMI categories were computed. Then, the neonatal outcomes for women with different BMI categories were contrasted. Characteristics of the cohort and neonatal outcomes were described using mean with standard deviation (SD) for continuous variables and number of cases with percentages for categorical variables. The between-group differences were tested using the one-way ANOVA test for continuous variables and the Pearson chi-square test for categorical variables. Then, we used logistic regression models with generalized estimating equations for clustering by patients to explore the effects of BMI on neonatal outcomes. The unadjusted and adjusted odds ratios (OR) and corresponding 95% confidence intervals (CIs) were reported. Maternal age, infertility type (primary or secondary infertility), infertility duration, parity (no child/one/two or more children), ART procedure type (IVF, ICSI, or mixed IVF or ICSI), embryo stage at transfer (day 3 or day 5/6), number of embryos transferred (1 or 2), infant gender (boy or girl), and year of birth were covariates adjusting for analyses. All statistical analyses were performed using the statistical package Stata, version 12 (StataCorp. Stata Statistical Software: Release 12. College Station, TX, USA) and a two-sided 5% level of significance.

## Results

A total of 1,676 live singleton births from mothers with PCOS after FET were enrolled in the study. Of these singletons, 101(6.02%) were delivered by mothers with underweight, 1,148 (68.50%) by mothers with normal weight, and 427(25.48%) by mothers with overweight. The basic characteristics of mothers across BMI categories are shown in Table 1. The underweight mothers were younger and had a shorter duration of infertility. However, the distribution in infertility type, parity, fertilization methods, and the number or developmental stage of the embryo at transfer showed no significant difference between BMI categories. IVF was the main fertilization method and about 80% of FET cycles were carried out with two embryos to transfer at day 3 in all the three BMI categories.

**Table 1:** Characteristics of the frozen-thawed embryo transfer cycles by maternal pre-pregnancy BMI among women with PCOS.

	**Underweight, *n* (%)** ** (BMI < 18.5 kg/m^2^)**	**Normal weight, *n* (%)** ** (BMI 18.5–24.9 kg/m^2^)**	**Overweight, *n* (%)** ** (BMI 25.0–29.9 kg/m^2^)**	***P*-value**
Overall	101	1,148	427	
Maternal age (years), mean±SD	28.82 ± 2.89	30.10 ± 3.47	30.48 ± 3.56	<0.001
BMI^*^ (kg/m^2^), mean±SD	17.61 ± 0.79	21.82 ± 1.76	26.99 ± 1.39	<0.001
Infertility duration (years), mean±SD	3.74 ± 2.28	3.20 ± 1.67	4.22 ± 2.54	<0.001
**Infertility type**				0.440
Primary infertility	68 (67.33)	712 (62.02)	275 (64.40)	
Secondary infertility	33 (32.67)	436 (37.98)	152 (35.60)	
**Parity**				0.728
0	99 (98.02)	1,110 (96.69)	410 (96.02)	
1	2 (1.98)	36 (3.14)	17 (3.98)	
≥2	0	2 (0.17)	0	
**Type of ART procedure**				0.196
IVF	60 (59.41)	676 (58.89)	259 (60.66)	
ICSI	15 (14.85)	259 (22.56)	84 (19.67)	
Mixed IVF and ICSI	26 (25.74)	213 (18.55)	84 (19.67)	
**Number of embryos transferred**				0.052
1	16 (15.84)	183 (15.94)	90 (21.08)	
2	85 (84.16)	965 (84.06)	337 (78.92)	
**Embryo stage at transfer**				0.157
Day 3	81 (80.20)	967 (84.23)	372 (87.12)	
Day 5/6	20 (19.80)	181 (15.77)	55 (12.88)	
**Year of birth**				0.303
2007–2012	8 (7.92)	95 (8.28)	29 (6.79)	
2013–2015	34 (33.66)	400 (34.84)	129 (30.21)	
2016–2019	59 (58.42)	653 (56.88)	269 (63.00)	

*PCOS*, polycystic ovary syndrome; *BMI*, body mass index; *ART*, assisted reproductive technology; *IVF, in vitro* fertilization; *ICSI*, intracytoplasmic sperm injection.

* The body mass index is the weight in kilograms divided by the square of the height in meters.

The neonatal outcomes according to maternal pre-pregnancy BMI are shown in Table 2. The percentage of male and female infants was similar across three BMI categories. The mean gestational age increased significantly with the increased maternal BMI. However, no marked difference was found in the rate of preterm birth among the three categories. The mean birth weight was 3,192.92, 3,300.39, and 3,369.45 g for infants from underweight mothers, normal-weight mothers, and overweight mothers, respectively, reaching significant differences between the three categories. When compared to infants from mothers with normal weight, the probability of high birth weight was significantly higher for infants from overweight mothers (9.13 vs. 5.49%); the risk of SGA infant significantly increased among underweight mothers (9.90 vs. 4.36%). Similarly, the rate of LGA infants was lower for underweight mothers (7.92%) and higher for overweight mothers (25.06%) when compared with normal-weight mothers (15.94%).

**Table 2:** Neonatal outcomes according to pre-pregnancy BMI among women with PCOS following frozen-thawed embryo transfers.

	**Underweight, *n* (%)** ** (BMI < 18.5 kg/m^2^)**	**Normal weight, *n* (%)** ** (BMI 18.5–24.9 kg/m^2^)**	**Overweight, *n* (%)** ** (BMI 25.0–29.9 kg/m^2^)**	***P*-value**
Overall	101	1,148	427	
Male gender	50 (49.50)	586 (51.05)	202 (47.31)	0.408
Gestational age (week), mean±SD	38.55 ± 1.25	38.35 ± 1.81	38.03 ± 2.10	0.003
Very preterm (<32 weeks)	0	16 (1.39)	9 (2.11)	0.333
Preterm (<37 weeks)	6 (5.94)	110 (9.58)	50 (11.71)	0.176
Birth weight (g), mean±SD	3,192.92 ± 411.04	3,300.39 ± 525.38	3,369.45 ± 597.11	0.006
Low birth weight (<2,500 g)	4 (3.96)	62 (5.40)	26 (6.09)	0.752
High birth weight (> 4,500 g)	4 (3.96)	63 (5.49)	39 (9.13)	0.023
Small for gestational age (SGA)	10 (9.90)	50 (4.36)	14 (3.28)	0.014
Appropriate for gestational age (AGA)	83 (82.18)	915 (79.70)	306 (71.66)	0.002
Large for gestational age (LGA)	8 (7.92)	183 (15.94)	107 (25.06)	<0.001

*PCOS*, polycystic ovary syndrome; *BMI*, body mass index (calculated as weight in kilograms divided by height in meters squared). Preterm birth was defined as delivery occurring before 37 weeks of gestation.

**Figure 1:**
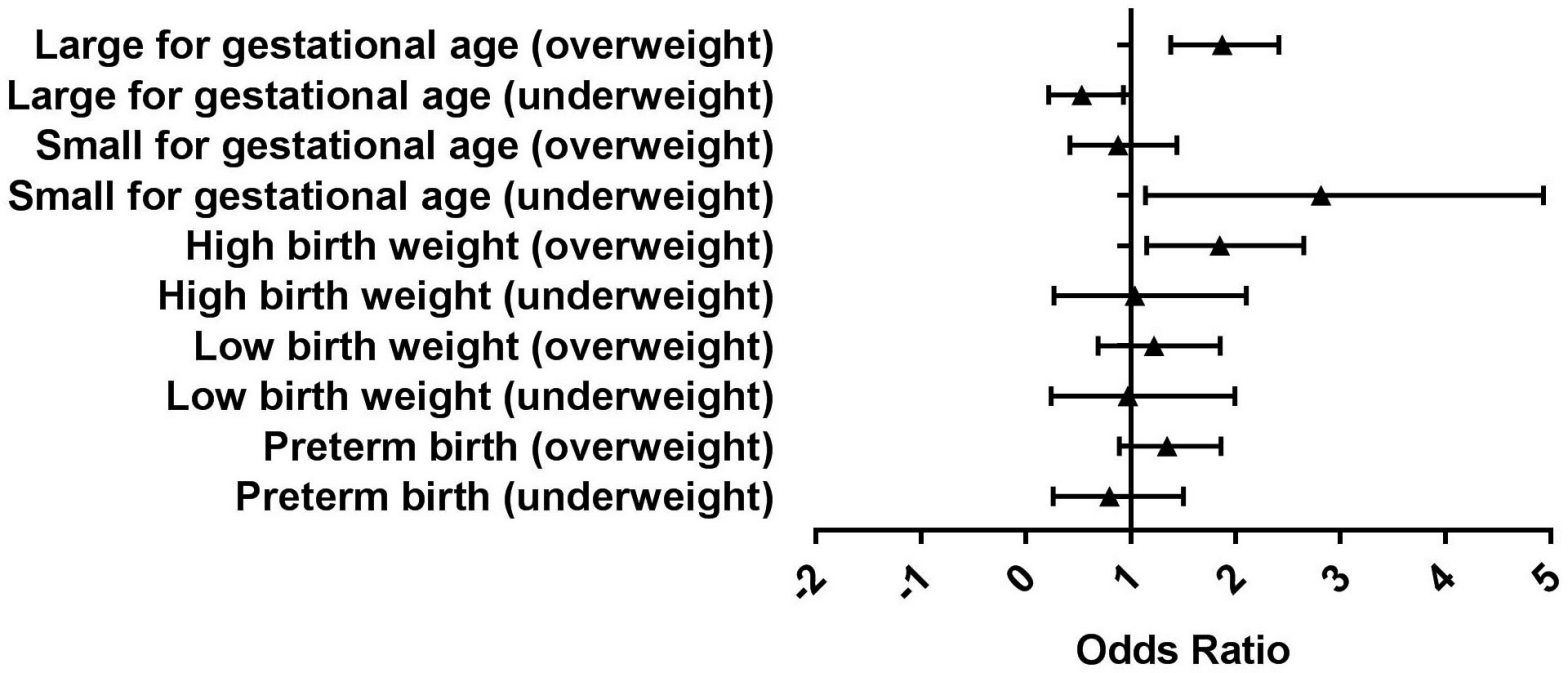
Adjusted odds ratios (ORs) of adverse neonatal outcomes among singletons with different maternal body mass index (BMI).

The logistic regression analyses were used to explore the effect of pre-pregnancy BMI on neonatal outcomes (Table 3, Figure 1). The risk of high birth weight for infants born from overweight mothers (adjusted odds ratio [aOR] 1.75, 95% confidence interval [95%CI] 1.15–2.65) was significantly higher than those from normal-weight mothers after adjusting for known confounding factors. The risk of SGA singleton (aOR 2.37, 95%CI 1.14-4.93) was higher among underweight mothers when compared to normal-weight mothers. Compared with normal-weight mothers, the risk of LGA babies (aOR 0.45, 95%CI 0.22–0.93) significantly decreased for underweight mothers and significantly increased (aOR 1.82, 95%CI 1.38–2.41) for overweight mothers. There was no statistically significant difference in the risk of preterm birth and low birth weight between different BMI categories.

**Table 3:** ORs (95%CI) for the relationship of pre-pregnancy BMI with neonatal outcomes among women with PCOS following frozen-thawed embryo transfers.

	**Underweight** ** (BMI < 18.5 kg/m^2^)**	**Normal weight** ** (BMI 18.5–24.9 kg/m^2^)**	**Overweight** ** (BMI 25.0–29.9 kg/m^2^)**
**Preterm (<37 weeks)**			
Unadjusted OR (95%CI)	0.60 (0.25, 1.39)	Ref	1.25 (0.88, 1.78)
Adjusted OR (95%CI)^a^	0.63 (0.26, 1.50)	Ref	1.29 (0.89, 1.86)
**Low birth weight (<** **2,500 g)**			
Unadjusted OR (95%CI)	0.72 (0.26, 2.03)	Ref	1.14 (0.71, 1.82)
Adjusted OR (95%CI)^a^	0.69 (0.24, 1.99)	Ref	1.13 (0.69, 1.85)
**High birth weight (>** **4,500 g)**			
Unadjusted OR (95%CI)	0.71 (0.25, 1.98)	Ref	**1.73 (1.14, 2.62)**
Adjusted OR (95%CI)^a^	0.75 (0.27, 2.10)	Ref	**1.75 (1.15, 2.65)**
**Small for gestational age**			
Unadjusted OR (95%CI)	**2.41 (1.18, 4.93)**	Ref	0.74 (0.41, 1.36)
Adjusted OR (95%CI)^a^	**2.37 (1.14, 4.93)**	Ref	0.78 (0.42, 1.44)
**Large for gestational age**			
Unadjusted OR (95%CI)	**0.45 (0.22, 0.94)**	Ref	**1.76 (1.34, 2.31)**
Adjusted OR (95%CI)^a^	**0.45 (0.22, 0.93)**	Ref	**1.82 (1.38, 2.41)**

*ORs*, odds ratios; *PCOS*, polycystic ovary syndrome; *BMI*, body mass index. All models included generalized estimating equations to account for clustering by the patient.

a Maternal age, primary infertility, parity, infertility duration, type of ART procedure, number of embryos transferred, embryo stage at transfer, offspring gender, and year of birth were adjusted for models.

Note: Results showed in bold indicated statistically significant associations.

## Discussion

This study focused on the effect of pre-pregnancy BMI on neonatal outcomes for singletons from mothers with PCOS following FET, and the result showed that maternal underweight was a significant protective factor against LGA infants for singletons born from patients with PCOS after FET, whereas maternal overweight was an adverse factor for LGA infant. In addition, the risk of high birth weight was significantly higher for infants from overweight mothers, and the risk of SGA babies was higher for infants from underweight mothers, compared with infants from normal-weight mothers.

Excessive fetal growth can result in LGA infant or fetal macrosomia, which substantially raises the risk of short and long-term morbidities and mortality (21–24). In the short term, LGA infant or fetal macrosomia increases the difficulties in delivery, which can lead to hypoxic brain damage or even stillbirth (21). In the long term, infants with LGA or macrosomia are predisposed to developing obesity, type 2 diabetes, and heart disease in adulthood (22–24). Pre-pregnancy BMI is a potentially modifiable and preventable lifestyle-related factor related to neonatal outcomes, and several research studies have been done to explore the influence of pre-pregnancy BMI on neonatal birth weight (25, 26). The majority of studies on this subject were completed in the general population and did not consider the effect of the type of conception and specific features of patients with PCOS (27, 28). In a meta-analysis including 30 studies, Gaudet et al. (27) reported that the risk of delivering an LGA infant increased by 142%, the risk of delivering a baby with birth weight ≥ 4,000 g increased by 117%, and the risk of delivering a baby with birth weight ≥ 4,500 g increased by 277% for obese mothers. Although several research studies focusing on BMI and birth weight were performed among women with PCOS, the results were contradictory, and the risk factors contributing to high birth weight including infertility treatment were not included (4, 14). There are also a few studies exploring the association of BMI with birth weight in patients with ART but did not reach a consistent conclusion (29, 30). Kawwass et al. (29) reported that obese status was related to an increased risk of low birth weight neonates among women with singleton pregnancies undergoing ART. According to a retrospective cohort study performed by Ben-haroush et al. (30) among patients with single embryo transfers, there were no significant differences in mean birth weight and proportion of LGA infants between singletons born from mothers with normal weight, overweight, and obese. However, these above studies were all conducted in fresh embryo transfer cycles which could not avoid the harmful effect caused by the supraphysiologic hormonal milieu or suboptimal endometrial development during controlled ovarian hyperstimulation (COH) and did not distinguish patients with PCOS from those with non-PCOS. For women with PCOS undergoing ART treatment, few research studies have been carried out to investigate the relationship between maternal BMI and adverse birth weight. Our study further enriched the currently existing literature by finding that the probability of delivering a baby with high birth weight or delivering an LGA baby significantly increased for PCOS women with overweight and pregnant after FET. Although several studies explained that the dysregulation of glucose, insulin, lipid, and amino acid metabolism, and the potentially pathological and adaptive response of the placenta to maternal increased BMI, these factors probably play a certain role in the influence of maternal overweight and increased infant birth weight, and further research is needed to elucidate the underlying mechanisms (31–34).

Our findings suggested that pre-pregnancy underweight increased the risk of SGA infants. A similar result was reported by Zhang et al. (35) exploring the relationship between maternal BMI and SGA in a population-based cohort study with 76,695 live births. They also evaluated if the above association was modified by preterm birth status and reported that being underweight was not related to the risk of preterm SGA, but was related to the higher risk of term SGA. We cannot confirm this result by the stratified analysis because of the small sample size for infants with SGA. In addition, a systematic review and meta-analysis by Liu et al. (28) including 46 population-based cohort studies in China also revealed that maternal underweight was related to an increased risk of SGA. In contrast with the previous study finding that pre-pregnancy underweight was not significantly related to LGA, our results suggested that pre-pregnancy underweight was a protective factor against LGA (25).

Preterm birth is related to a high risk of neonatal mortality and morbidity (36). Survivors of preterm birth have a higher risk of neurodevelopmental problems and long-term disability (37). A comprehensive systematic review by McDonald et al. (38) included 84 studies with 1,095,834 women from both developed countries and developing countries and showed that overweight women had a higher probability of having an infant of preterm birth. Han et al. (39) reported a positive association between preterm birth and underweight in developed countries, but no significant association was found in developing countries by performing a systematic and comprehensive summary of all studies between 1950 and 2009 including 78 studies and 1,025,794 women in developing and developed countries. However, there was no significant association between pre-pregnancy BMI and preterm among women with PCOS getting pregnant after FET, which was inconsistent with results from previous studies. Further research from large prospective studies to verify this finding is needed.

The main strength of this study was that we concentrated exclusively on singletons born from women with PCOS conceived by FET. The finding of this study offered supporting evidence for evaluating the effect of pre-pregnancy BMI on neonatal outcomes. The dataset obtained from the single center avoided the potential interference from different ART techniques and laboratory environments in centers on neonatal outcomes. However, the study has several limitations. The first inherent limitation of this study was the retrospective design; however, strict inclusion criteria have been set to ensure high-quality data. The second limitation was that we did not explore the association between obesity and adverse neonatal health in this study owing to the small number of obese women with PCOS. The third limitation was that some potential confounders could not be adjusted including maternal nutrition conditions, educational status, and the type of PCOS. Finally, no long-term follow-up was conducted among offspring from mothers with PCOS, thus great attention should be devoted to the growth, development, and psychosocial health of offspring in the future.

In conclusion, this study provided scientific evidence that maternal pre-pregnancy BMI affected the birth weight of singletons born from mothers with PCOS after FET cycles. For women with PCOS getting pregnant by FET, being overweight was a risk factor for having an LGA baby, whereas being underweight was a protective factor for having an LGA baby. In addition, being overweight was significantly associated with an increased risk of high birth weight, and being underweight was related to an increased risk of SGA. These findings are vital for clinical practice. Clinicians should encourage women with PCOS to optimize their BMI before fertility treatment by taking some measures including dietary adjustment, physical exercise, and behavioral intervention. Maternity and newborn care providers should pay more attention to women with PCOS with abnormal BMI for the prevention of adverse infant outcomes.

## Data availability statement

The raw data supporting the conclusions of this article will be made available by the authors, without undue reservation.

## Ethics statement

The studies involving human participants were reviewed and approved by the Ethics Committee (Institutional Review Board) of Shanghai Ninth People's Hospital Affiliated to Jiao Tong University School of Medicine. The patients/participants provided their written informed consent to participate in this study.

## Author contributions

QZ and HGu designed the study and drafted the manuscript. HGa and BW contributed to data collection and analysis. QZ revised the article. All authors read and approved the final manuscript.

## Funding

This study was funded by the National Natural Science Foundation of China (Grant Nos. 81903324, 82273634) and the interdisciplinary program of Shanghai Jiao Tong University (YG2019QNA19).

## Conflict of interest

The authors declare that the research was conducted in the absence of any commercial or financial relationships that could be construed as a potential conflict of interest.

## Publisher's note

All claims expressed in this article are solely those of the authors and do not necessarily represent those of their affiliated organizations, or those of the publisher, the editors and the reviewers. Any product that may be evaluated in this article, or claim that may be made by its manufacturer, is not guaranteed or endorsed by the publisher.
